# Effects of Exogenous Tryptophan in Alleviating Transport Stress in Pearl Gentian Grouper (*Epinephelus fuscoguttatus* ♀ × *E. lanceolatus* ♂)

**DOI:** 10.3390/ani14243583

**Published:** 2024-12-12

**Authors:** Jie Cao, Dan Fang, Weiqiang Qiu, Jing Xie

**Affiliations:** 1 College of Food Science and Technology, Shanghai Ocean University, Shanghai 201306, China; d220300072@st.shou.edu.cn (J.C.); m210300819@st.shou.edu.cn (D.F.); 2 National Experimental Teaching Demonstration Center for Food Science and Engineering, Shanghai Ocean University, Shanghai 201306, China; 3 Shanghai Engineering Research Center of Aquatic Product Processing and Preservation, Shanghai 201306, China; 4 Key Laboratory of Aquatic Products High-Quality Utilization, Storage and Transportation (Co-Construction by Ministry and Province), Ministry of Agriculture and Rural Affairs, Shanghai 201306, China

**Keywords:** tryptophan, grouper, transport stress, oxidative stress, apoptosis

## Abstract

Live fish transportation plays a crucial role in the commercial fish trade. Consequently, mitigating stress during transportation is essential for enhancing the survival rate of fish and reducing potential financial losses. In this study, the effectiveness was evaluated of exogenous tryptophan in reducing transport stress in hybrid grouper, *Epinephelus fuscoguttatus* ♀ × *E. lanceolatus* ♂. Firstly, the groupers were divided into the following five experimental groups: 40 mg/L MS-222 group, 30 mg/L tryptophan, 50 mg/L tryptophan, 70 mg/L tryptophan, and the control group without additives. Followed by transportation simulation, the fish samples were collected before and after transportation for the determination of antioxidant enzyme activities, apoptosis gene, and inflammatory gene expressions. The results indicated that the superoxide dismutase (SOD), catalase (CAT), and glutathione peroxidase (GSH-Px) activities and malondialdehyde (MDA) levels in all groups were significantly increased, while they were lower in the 50 mg/L Trp treated group compared to the control group (*p* < 0.05). Compared with the control group in the 50 mg/L Trp, 70 mg/L Trp, and 40 mg/L MS-222-treated groups, serum cortisol and blood glucose levels were significantly increased (*p* < 0.05), and anti-inflammatory factor (*IL-10*) gene expression was upregulated and pro-inflammatory factor (*IL-1β*) gene expression was decreased (*p* < 0.05). In addition, it was found that the 30 mg/L Trp, 50 mg/L Trp, and 40 mg/L MS-222 treatment groups had less green fluorescence than the control group by measuring the mitochondrial membrane potential, and 50 mg/L Trp and MS-222 showed more red fluorescence in fluorescence images than the other samples at the same sampling time. Therefore, in this study, it was demonstrated that the tryptophan could be used as a new anti-stress agent for hybrid groupers during transport, and additional research is required to identify the specific conditions that yield the best outcomes.

## Simple Summary

In this study, we added different concentrations of tryptophan during grouper transportation. Fish samples and water quality were examined after 72 h of transportation. The results showed that the tryptophan supplementation helped prevent increases in cortisol and blood glucose levels in fish transport stress. The addition of exogenous tryptophan to transport water may help to alleviate transport stress by improving water quality and depressing the enhancement of stress-related hormones.

## 1. Introduction

Shipping live fish is a critical component of the commercial fish industry. Throughout this process, fish encounter numerous stressors such as fasting, handling, vibrations, overcrowding, and deteriorating water quality. These factors can lead to metabolic and physiological imbalances, weakened immune systems, and ultimately, high mortality rates [1]. Given the significant economic value of the live commercial fish trade, the issues of fish welfare and the economic impact of transport-related losses have become a growing focus for researchers [2].

A series of measures have been taken to prevent the deterioration in the water and to reduce transportation stresses. The most common method is to use anesthetics to reduce the respiratory metabolic activities of fish during transportation. Nevertheless, some synthetic anesthetics have been revealed to have adverse side effects, such as excessive mucus production, gill irritation, suppression of the immune system, and increased muscle tension [3,4,5,6]. Nowadays, plant essential oils are studied as fish anesthetics. However, due to different extraction methods, even the same plant often yields inconsistent results in the extracts obtained. The efficacy and dosage of essential oils need to be confirmed in different species of fish before use [7].

Furthermore, reducing the physiological stress response which is under the control of the central nervous system is one way of reducing stress, such as neurotransmitters (Gamma-aminobutyric acid (GABA), guanidinoacetic acid (GAA), and 5-hydroxytryptamine (5-HT)) and some essential amino acids (phenylalanine (Phe) and tryptophan (Trp)) [8,9]. Supplementing fish diets with GABA, GAA, and 5-HT has been shown to have positive effects, including improved growth performance, increased antioxidant activity, and reduced aggression [10,11,12]. Tryptophan holds a critical position among the amino acids needed by fish, owing to its multifaceted functions. As an essential aromatic amino acid, tryptophan is indispensable for protein synthesis and also functions as a precursor for various compounds that exert multiple influences on fish, including their immune system, antioxidant capacity, stress levels, and behavioral reactions [13,14,15]. Given the complexity of tryptophan’s metabolic pathways and the significant roles its metabolites play, tryptophan is involved in numerous physiological functions [14]. The tryptophan serves as the precursor for the synthesis of various compounds, including 5-HT, the hormone melatonin, the amino acid kynurenine, and other related chemicals like kynurenic acid, quinolinic acid, and niacin [16]. 5-HT, a monoamine-based neurotransmitter, plays a role in modulating adaptive reactions and responses to environmental changes [17]. Tryptophan is the only precursor of 5-HT [18]. Consequently, it is essential for regulating various vertebrate functions, ranging across the neuroendocrine system to the immune system [19]. For instance, it has been shown that dietary tryptophan can counteract stress-induced transcriptional changes in teleost fish after inflammation [20]. There is another study showing tryptophan counteracting the effects of acute stress in *Salmo salar* [21]. Similarly, the stress-induced elevation of cortisol could also be counteracted by intaking tryptophan in *Oncorhynchus mykiss* [22].

Tryptophan could potentially serve as an effective agent for mitigating transport stress in fish; however, there has been no research conducted on its impact on fish transport stress. Therefore, in the current research, the aim was to investigate the impact of tryptophan on water quality indicators and stress biomarkers in fish, in order to assess its potential for mitigating the transportation stress.

## 2. Materials and Methods

### 2.1. Fish

Healthy pearl gentian groupers (450 g ± 3.5 g) were acquired from the Shanghai fishery market (Shanghai, China) and acclimated in the plastic tanks (2.4 × 1.7 × 0.6 m^3^, density: 50 g/L) for two weeks to adapt to the experimental environment. During the acclimatization period, the pearl gentian groupers were given two daily feedings of commercial floating pellets (30% protein, 5% lipid). All experimental fish were starved for 24 h before experiments. The parameters of water quality were kept within the following limits: salinity at 23.0 ± 1.0‰, dissolved oxygen at 7.0 ± 0.5 mg/L, temperature at 20 ± 0.5 °C, and pH at 7.0 ± 0.3.

### 2.2. Experimental Design

After two weeks of acclimatization, a total of 150 fish were randomly assigned to five different treatment groups (Table 1), which were subjected to 72 h transportation. The tryptophan concentrations and the MS-222 concentration used in this research were chosen based on the results of pre-experiments. When the concentration of MS-222 was 40 mg/L, the groupers were in a deep sedation period, which was suitable for transportation. Each experimental group was replicated three times, with each bag containing ten fish (fish to water ratio was 1:3). Then, the packed bags were pumped with pure oxygen, placed in a constant temperature shaker, and fish were simulated to be transported in a vibrating conveyor under 60 rpm at 15 ± 1 °C [23]. The water samples were collected at 0, 12, 24, 36, 48, 60, and 72 h. At the end of the transport, a random selection of three fish per treatment group was prepared for sampling [23].

### 2.3. Sampling

The pearl gentian grouper samples were anesthetized with 200 mg/L MS-222. Venous–arterial blood was sampled from caudal veins using a 2 mL syringe without anticoagulant and kept at 4 °C for a duration of 6 h while stationary [19]. The coagulated blood was centrifuged at 4000× *g* for 20 min at 4 °C. Subsequently, the dorsal muscles of pearl gentian groupers were obtained to determine the levels of metabolites. The liver tissues were excised for measuring mRNA expression. Samples were stored at −80 °C until use.

### 2.4. Measurement

#### 2.4.1. Water Quality Parameters

The DO level was determined by a JPSJ 605F dissolved oxygen meter (Yidian Scientific Instrument Co., Ltd., Shanghai, China); the TAN concentration was measured by a DWS-296 ammonia nitrogen meter (Yidian Scientific Instrument Co., Ltd., Shanghai, China); and the pH was measured by the pH meter (Testo 205, Detu Instruments International Trade Co., Shanghai, China).

#### 2.4.2. Antioxidant Enzyme Activity Analysis

The activities of serum SOD, CAT, GSH-Px, and MDA concentration were measured by commercial test kits (Nanjing Jiancheng Bioengineering Institute, Nanjing, China), as follows: SOD (A001-3–2, 96 T), CAT (A007-1–1, 100 T/96 S), GSH-Px (A005-1–2, 100 T/48 S), and MDA (A003-1–2, 100 T/96 S).

#### 2.4.3. Serum Biochemical Analysis

The serum glucose levels were determined using commercial assay kits (Nanjing Jiancheng Institute of Bioengineering, Nanjing, China), according to the manufacturer’s instructions. Serum cortisol was determined by means of an enzyme-linked immunoassay according to the cortisol ELISA kit manufacturer’s instructions (Shanghai Fanke Industrial Co., Ltd., Shanghai, China). Serum alanine aminotransferase (ALT) and aspartate aminotransferase (AST) activities were measured with ALT and AST kits (Nanjing Jiancheng Bioengineering Institute, Nanjing, China). Glucose (A154-1–1, 96 T); ALT (C009-1-1, 100 T/50 S); AST (C009-1-1, 100 T/50 S).

#### 2.4.4. Mitochondrial Membrane Potential Measurement

A total of 200 mg of liver was taken and rinsed with PBS, ground and added to 1.0 mL ice pre-cooled lysis buffer, repeatedly centrifuged to extract the supernatant, and finally 100 μL of Trypsin buffer was added to resuspend the mitochondrial precipitate. The changes in mitochondrial membrane potential were assessed employing the JC-1 assay kit. The purified 0.1 mL mitochondria were incubated with 0.9 mL JC-1 (10 µg/mL) staining solution for 20 min and observed using a confocal laser scanning microscope. Mitochondria exhibiting high membrane potential contained the JC-1 dye primarily in the form of red fluorescent aggregates within the matrix. In contrast, mitochondria with low membrane potential showed green fluorescence, indicating the presence of JC-1 in its monomeric form [20].

#### 2.4.5. Real-Time Quantitative PCR (qPCR)

Total RNA was extracted from the liver using the TRIzol reagent. The purity and concentration of extracted RNA were assessed using a NanoDropone spectrophotometer (NanoDrop Technologies, Wilmington, DE, USA). Single-stranded cDNA was generated from 1 μg of total RNA using the Servicebio^®^ RT cDNA Synthesis Kit, which includes gDNA Remover. The qPCR analysis was conducted using a 15 μL reaction system that comprised 2.0 μL of cDNA template, 1.5 μL of 2.5 μM gene primers (both forward and reverse), 7.5 μL of 2 × SYBR^®^ Green qPCR Master Mix, and RNase-free water (Servicebio Technology Co., Ltd., Wuhan, China) to make up the remainder. The cycling conditions were as follows: 30 s at 95 °C; then 40 cycles of 15 s at 95 °C and 30 s at 60 °C. GAPDH was used as a reference gene to normalize cDNA loading. The 2^−ΔΔCT^ method was used to calculate the gene expression levels [21]. The primers for qRT-PCR analyses were listed in Table 2.

### 2.5. Statistical Analysis

The normality of the data and the homogeneity of the variances were assessed and verified using the Shapiro–Wilk and Levene tests, respectively. One-way analysis of variance (ANOVA) was used to evaluate the anesthetic data utilized with SPSS Statistics 26.0 (SPSS, Chicago, IL, USA). The results are expressed as mean ± SD. Origin 2018 software was used to create graphs.

## 3. Results

### 3.1. Water Quality

**Figure 1:**
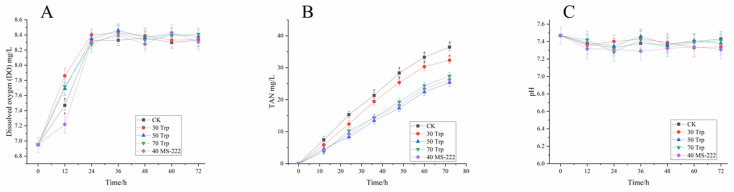
Changes in DO (**A**), TAN (**B**), and pH (**C**) in water during transportation. “*” indicates significant differences (*p* < 0.05) between the treatment groups.

Three fish deaths occurred in the CK group, and no mortality was observed in the Trp-treated groups and Ms-222 group during the transport. Figure 1 illustrates the changes in water quality parameters during transportation. In the CK group, the dissolved oxygen (DO) value was significantly reduced during transportation, all treatments with Trp and MS-222 had higher DO levels than the CK group after 72 h transport (*p* < 0.05). During the transport, a rise in TAN levels was observed across all groups, with higher concentrations in the CK and the 30 mg/L Trp groups (*p* < 0.05). By the end of transport, the water pH remained consistent across all treatments and matched the initial pH at 0 h (*p* > 0.05).

### 3.2. Oxidative Stress

**Figure 2:**
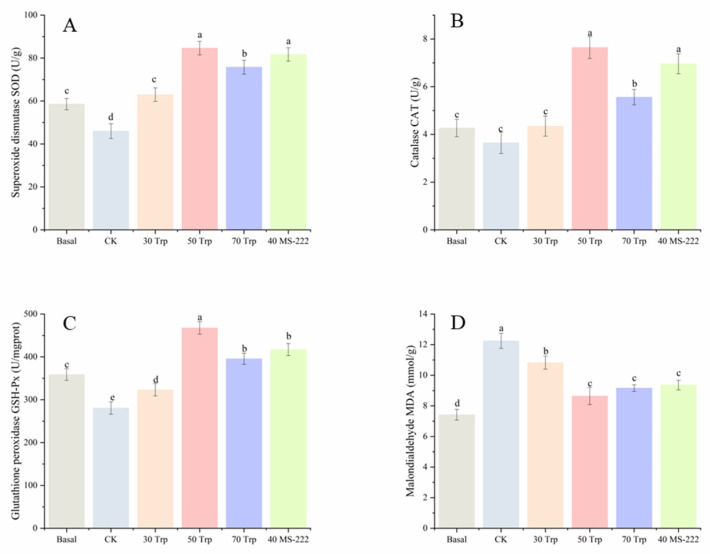
Oxidative stress parameters ((**A**), superoxide dismutase; (**B**), catalase; (**C**), glutathione reductase; (**D**), malondialdehyde) of pearl gentian grouper in each experimental group during the simulated transportation. Letters a~e indicate significant differences (*p*  <  0.05) between the treatment groups, the same below.

The serum SOD activity rose after transportation; the basal fish and treatments with Trp and MS-222 showed higher SOD activities than that of CK treatment (*p* < 0.05) (Figure 2A). Fish treated with 50 mg/L Trp exhibited higher serum CAT activity compared to CK, 30 mg/L Trp treatments, and basal fish (*p* < 0.05), but similar to the 40 mg/L MS-222 treatment (Figure 2B) (*p* > 0.05). The serum GPx activity dropped below the basal level in both the CK and 30 mg/L Trp groups (*p* < 0.05). Fish treated with 50 mg/L Trp showed elevated GPx activity compared to the basal and other treatment groups (Figure 2C) (*p* < 0.05). The CK group exhibited a higher MDA level compared to the basal and other treatment groups after transportation (Figure 2D) (*p* < 0.05).

### 3.3. Blood Biochemical Parameters

**Figure 3:**
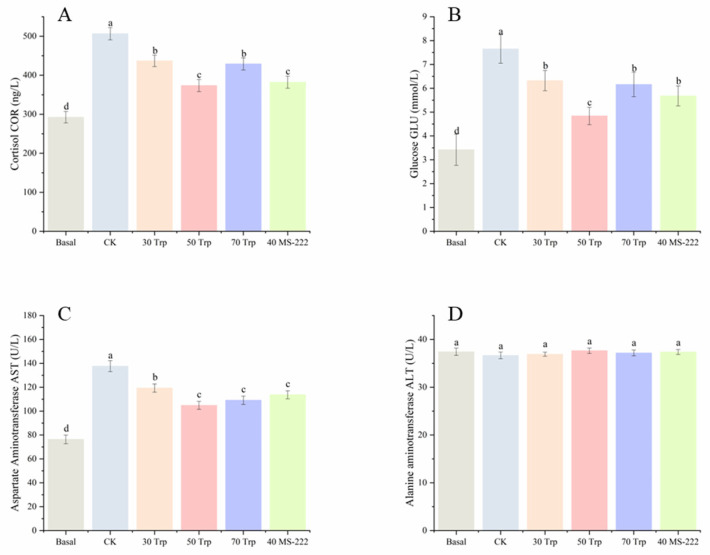
Serum biochemical parameters ((**A**), cortisol; (**B**), glucose; (**C**), aspartate transaminase; (**D**), alanine aminotransferase) of pearl gentian grouper in each experimental group during the simulated transportation. Letters a~d indicate signifcant diferences (*p* < 0.05) between the treatment groups”.

The fish treated with 50 mg/L Trp and 40 mg/L MS-222 showed lower cortisol level compared to the fish in the Ck group and other Trp treatment groups (*p* < 0.05) (Figure 3A). After 72 h of transport, the CK group showed elevated serum glucose levels compared to the basal group, while Trp treatment inhibited this rise (mainly with 50 mg/L Trp; *p* < 0.05) (Figure 3B). The fish transported with Trp and MS-222 showed lower AST activity compared to those of the CK treatment (Figure 3C) (*p* < 0.05). After 72 h of transport, no significant differences in ALT levels were observed (Figure 3D) (*p* > 0.05).

### 3.4. Mitochondrial Membrane Potential Analysis

**Figure 4:**
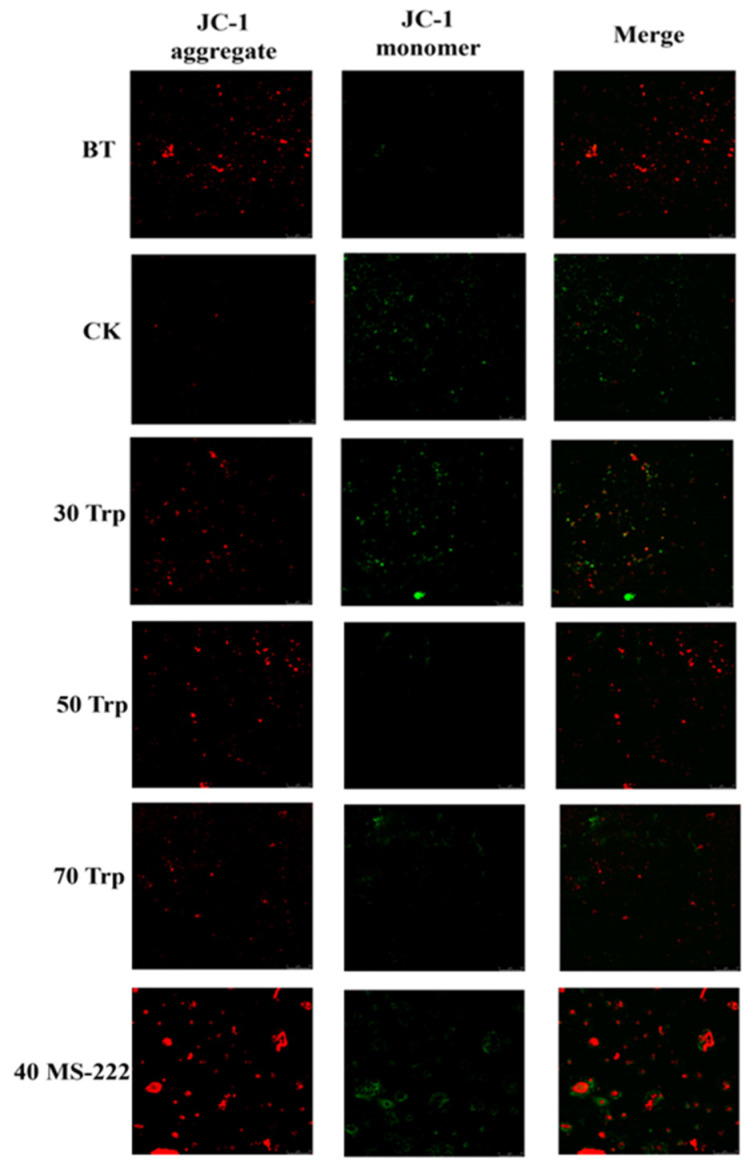
Mitochondrial confocal images of pearl gentian grouper transported in different treatment groups.

As shown in Figure 4, the confocal image of the livers of basal fish showed red fluorescence. The red fluorescence of the livers in the CK group decreased and the green fluorescence increased after 72 h of transport. Fish transported with 50 mg/L Trp and 70 MS-222 mg/L Trp showed less green fluorescence than those of fish in the CK, 30 mg/L Trp, and 40 mg/L MS-222 treatment groups.

### 3.5. Gene Expression

**Figure 5:**
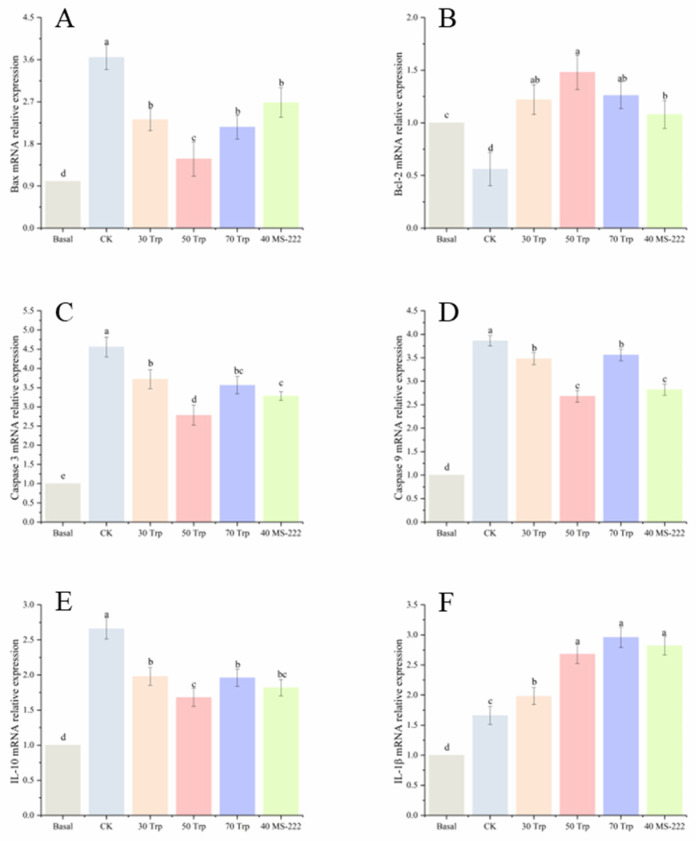
Apoptosis gene (**A**), *bax*; (**B**), *bcl-2*; (**C**) *caspase 3*; (**D**), *caspase 9*) and inflammatory gene (**E**), *IL-10*; (**F**), *IL-1β*) expressions of pearl gentian grouper transported in different treatment groups. Letters a~d indicate signifcant diferences (*p* < 0.05) between the treatment groups.

As shown in Figure 5, after 72 h of transport, the expression levels of *bax*, *caspase 3*, and *caspase 9* were increased. The CK group exhibited notably higher expression levels of *bax*, *caspase 3*, and *caspase 9* compared to the fish treated with tryptophan and MS-222 (Figure 5A,C,D) (*p* < 0.05). Conversely, the expression of B-cell lymphoma-2 (*bcl-2*) in the CK group was lower compared to the tryptophan and MS-222-treated fish (Figure 5B) (*p* < 0.05).

The expression of interleukin-10 (*IL-10*) was higher in the fish transported with Trp and MS-222 than the basal fish but lower than the CK fish (Figure 5E) (*p* < 0.05). Fish treated with 50 mg/L Trp and 70 mg/L Trp showed a higher expression of interleukin-β (*IL-β*) than the basal fish and the fish in the CK and 30 mg/L Trp treatment groups (Figure 5F) (*p* < 0.05).

## 4. Discussion

The shipping of live fish plays a vital role in the economic fish trade industry. The transportation can induce a significant stress response in fish, potentially leading to substantial economic losses [2]. Typically, the stress response during fish transport is regulated through hormonal pathways, the hypothalamic–pituitary–inter-renal (HPI) axis [24]. The primary hormonal reaction involves the secretion of cortisol by the adrenal cortex in the head kidney. The level and degree of cortisol elevation are directly correlated with the severity and duration of the stressors [25]. The results revealed that the content of cortisol increased after 72 h of transport. The content of cortisol was significantly lower in 50 mg/L Trp and 40 mg/L MS-222 treatment groups than other groups after transport, which could indicate an adaptation to the novel environment. Moreover, cortisol release is controlled through negative feedback mechanisms on the hypothalamic–pituitary–inter-renal (HPI) axis, and elevated cortisol levels can decrease target tissue sensitivity by reducing receptor numbers [26].

An increase in blood glucose levels is viewed as a secondary stress reaction in fish, stemming from gluconeogenesis and glycogenolysis processes [27]. Barton carried out a comparative analysis to examine how four different salmon species reacted to identical handling and transport stressors [28]. The findings indicated that the species perceived as most stressed varied depending on whether cortisol or glucose levels were used as the stress indicator, highlighting that stress assessment should not be based on a single parameter. Wu et al. found that *G. tomentella* supplementation decreased plasma cortisol in transported koi carp, yet it had no effect on glucose levels [29]. Our study also found that cortisol levels were more responsive to added substances than glucose levels after the transport process.

AST and ALT serve as biomarkers for tissue injury and participate in the gluconeogenic process, which liberates amino acids to synthesize glucose [30]. Consequently, the elevated AST activities observed in the CK group could be linked to the increased glucose production which required aspartate, given that this treatment also presented the highest glucose concentrations [31].

Maintaining good water quality in transport is vital for fish health and survival, thus helping reduce stress. Throughout the transportation process, the ammonia levels keep accumulating because fish metabolites are not being removed. Ammonia can harm various fish tissues, such as the gills, liver, kidneys, and nervous system [32]. It has been reported that the common carp exposed to sublethal doses of ammonia has led to increased adrenaline levels [32]. Typically, it is advised to fast fish for at least 24 h before transport to lessen fecal waste [33]. CO_2_ is an important metabolic byproduct of fish during transportation. The accumulation of CO_2_ can lead to acidosis in fish, causing narcosis and death [34]. In our studies, as the transport duration extended, the water became increasingly turbid and ammonia nitrogen levels rose. However, the DO remained fairly stable, with no oxygen depletion observed after 72 h of transport, indicating that oxygen levels were not a constraint in the sealed, oxygen-enriched transport setup. This was in agreement with an earlier study of an earlier study [8]. At present, there is a scarcity of research documenting the oxidative stress in fish associated with oxygen supersaturation during transportation.

Reactive oxygen species (ROS) are generated within the mitochondria and damage the mitochondrial components [35]. A cytotoxicity test that assesses mitochondrial respiration could detect initial indications of cytotoxicity after exposure to mitochondrial toxicant [36]. After transport, we observed a significant decrease in the mitochondrial membrane potential in CK groupers, which revealed a possible mechanism for transport stress-induced toxicity, where such stress impairs mitochondrial function, resulting in elevated levels of intracellular ROS. Our findings further indicate that prolonged transport stress (72 h) results in heightened ROS levels and initiates apoptosis via the mitochondrial pathway.

Antioxidant enzymes are vital in combating oxidative stress [37,38]. The SOD preferentially removes superoxide anion radicals to generate H_2_O_2_ [39]. Then, the CAT and GSH-Px will break down the H_2_O_2_ into water [40]. Our results showed that serum SOD, CAT, and GSH-Px activities decreased in the CK group, which may contribute to prolonged transportation stress. Hong et al. discovered that the activities of SOD and CAT reduced in the liver of rachinotus ovatus after 8 h of transportation [41]. Ren et al. also found inhibited activities of SOD and CAT and increased MDA content in rainbow trout during transportation. These findings suggest that extended transportation inhibited the balance of antioxidant in fish. The increase in serum SOD levels observed in grouper following transport could be attributed to the body’s response to oxidative stress, which was sufficient to prevent a rise in ROS levels. The increase in serum SOD levels observed in grouper following transport could be attributed to the body’s response to oxidative stress, which was sufficient to prevent a rise in ROS levels. The elevation of serum GPx in fish treated with 50 mg/L tryptophan and 40 mg/L MS-222 might be the organism’s way of countering the increased presence of oxidized substances within cells. Notably, this was the sole cellular damage indicator that did not decrease with the application of these substances. MDA is an important marker of lipid peroxidation [42]. MDA levels offer significant information about the degree of lipid peroxidation in cell membranes, thereby indirectly reflecting the extent of cell membrane damage [42]. The CK group exhibited a significant increase in MDA content following transport, consistent with mitochondrial membrane potential findings, indicating that the simulated transport induced oxidative stress leading to cell membrane damage.

An excess of ROS hastens cellular damage and triggers inflammation [43]. Inflammation is crucial for promoting tissue repair, removing damaged tissue, and safeguarding the host from stress [44]. NF-κB is a crucial transcriptional factor in inflammatory processes and can be triggered by various extracellular signals in different inflammatory pathways [45]. When faced with unfavorable conditions, NF-κB is activated by separating from IκB and promotes the expression of proinflammatory cytokines, such as *IL-1β*, *IL-6*, and *IL-8* [38]. They can mediate various immune responses characterized by the induction of the inflammatory response [46]. Conversely, anti-inflammatory cytokines (e.g., *IL-10* and *TGF-β1*) are responsible for eliminating the inflammatory response and restoring the organism to normality. Bai et al. showed that transport stress caused an increase in the expression levels of *IL-1β* in *Pelteobagrus fulvidraco* [47]. Hoseini et al. also found that *IL-1β* expression levels were upregulated in carp under transport stress [48]. In this study, the *IL-1β* gene in the CK group showed the highest expression after transport, suggesting that the transport stress triggered an inflammatory response in the organism. This is consistent with the findings of Christopher et al. who found significant upregulation of *IL-1β* expression in *Gadus morhua* following overcrowding [49]. Meanwhile, the fish transported with 50 mg/L Trp and 40 mg/L MS-222 inhibited the increase in *IL-1β* expression and had no effect on the *IL-10* expression.

Furthermore, oxidative stress not only triggers inflammation but also induces cell apoptosis [38]. Most apoptotic signaling processes are associated with the altered expression of apoptotic genes such as *Bax*, *Bcl-2*, and the caspase family. *Bax* is a pro-apoptotic factor, whereas *Bcl-2*, in contrast, inhibits apoptosis [50]. Bax facilitates the release of cytochrome C into the cytoplasm, while the anti-apoptotic protein Bcl-2 prevents the release of cytochrome C from the mitochondria [51]. Changes in the intracellular ratio of *Bcl-2* and *Bax* expressions influence the release of mitochondrial cytochrome C, leading to caspase activation; this also plays a crucial role in the mediation of apoptosis [52]. *Caspase 3* acts as the key executioner molecule, while *Caspase 9* serves as the pivotal initiator molecule [53]. In this study, it was demonstrated that transport stress elevated the expression levels of *Bax*, *Caspase 3*, and *Caspase 9*, suggesting that the caspase-dependent apoptotic pathway might be involved in the apoptosis of the pearl gentian grouper. Furthermore, fluctuations in the mitochondrial membrane potential and the subsequent release of cytochrome C from the mitochondria, due to heightened ROS levels, have been shown to result in the activation of *Caspase 3* in As_2_O_3_-treated mammalian cells [54]. This could also be the reason for the caspase activation.

## 5. Conclusions

In this study, the use of tryptophan could show a positive effect in reducing transport stress in *Epinephelus fuscoguttatus* ♀ ×* E. lanceolatus* ♂ compared to no additives. Tryptophan supplementation prevented increases in cortisol and blood glucose levels in fish transport stress. Furthermore, a dosage of 50 mg/L tryptophan provided superior protection against oxidative stress compared to other treatments. Consequently, for the transportation of *E. fuscoguttatus* ♀ ×* E. lanceolatus* ♂, while the tryptophan showed positive outcomes, further research is needed to assess its impact on the general health of fish during and post-transportation, as well as on the quality of the flesh.

## Figures and Tables

**Table 1:** Experimental grouping.

Groups	Additives
Basal	No transportation; no additives
Control (CK)	Transport; no additives
30 Trp	Transport; 30 mg/L Tryptophan
50 Trp	Transport; 50 mg/L Tryptophan
70 Trp	Transport; 70 mg/L Tryptophan
40 MS-222	Transport; 40 mg/L MS-222

**Table 2:** Primer sequence for quantitative PCR.

Groups	Additives	Amplification Efficiency (%)	bp
*GAPDH*	F: CATCACTGCCACCCAGAAGA R: GACAGCTTTAGCAGCACCAGTAGA	98.7	293
*Bcl-2*	F: ATCGTAGGGCTTTTCGCTTTC R: CTCCCATCCTCTTTGGCTCTG	95.5	235
*Bax*	F: ACTGGGGAAGAATCATCGTG R: CGTCCTGAAGAAATCCAAACA	96.7	155
*Caspase 3*	F: CGCAAAGAGTAGCGACGGA R: CGATGCTGGGGAAATTCAGAC	94.1	106
*Caspase 9*	F: TTTTCCTGGTTATGTTTCGTGG	96.9	135
	R: TTGCTTGTAGAGCCCTTTTGC		
*IL-10*	F: AAGCAAACGACGACTTGGACAC R: TTAGATTCCTGGTATCCTCCGTC	94.2	249
*IL-1β*	F: ATGAAAGTCTGACCGTCCTCCTG R: AACACGGCTTTGTCGTCTTTC	95.6	118

## Data Availability

The original contributions presented in this study are included in the article; further inquiries can be directed to the corresponding authors.
